# Hsa_circ_0004712 downregulation attenuates ovarian cancer malignant development by targeting the miR-331-3p/FZD4 pathway

**DOI:** 10.1186/s13048-021-00859-0

**Published:** 2021-09-10

**Authors:** Xuan Zhou, Jinchi Jiang, Shuaishuai Guo

**Affiliations:** 1 Reproductive Medicine Center, Shenyang Women’s and Children’s Hospital, No. 87 Danan Street, Shenhe District, Shenyang, 110000 Liaoning China; 2 Department of Radiology, Shenyang Women’s and Children’s Hospital, Shenyang, Liaoning China

**Keywords:** hsa_circ_0004712, miR-331-3p, FZD4, Ovarian cancer

## Abstract

**Background:**

Circular RNAs (circRNAs) are gradually reported to be implicated in the development of malignant tumors, including ovarian cancer (OC). This paper intended to explore the function and action mechanism of hsa_circ_0004712 in OC.

**Results:**

In our results, hsa_circ_0004712 was aberrantly overexpressed in OC tissues and cells. Downregulation of hsa_circ_0004712 impaired OC cell proliferation, colony formation, invasion and migration, and accelerated apoptosis. Hsa_circ_0004712 directly targeted miR-331-3p whose inhibitors reversed the effects of hsa_circ_0004712 downregulation. FZD4 was targeted by miR-331-3p, and hsa_circ_0004712 could positively regulated FZD4 expression by targeting miR-331-3p. The anti-tumor effects of miR-331-3p restoration were reversed by FZD4 overexpression. Downregulation of hsa_circ_0004712 also impaired tumor development in vivo by regulating miR-331-3p and FZD4.

**Conclusion:**

In conclusion, hsa_circ_0004712 deficiency repressed OC development by mediating the miR-331-3p/FZD4 pathway, predicting that hsa_circ_0004712 was a promising biomarker for OC diagnosis and therapy.

**Supplementary Information:**

The online version contains supplementary material available at 10.1186/s13048-021-00859-0.

## Introduction

Ovarian cancer (OC) is recognized as the most common and deadly gynecological cancer in women [1, 2]. The primary form of OC is epithelial ovarian cancer (EOC), which accounts for more than 90% of OC cases [3]. Studies show that more than 70% of OC cases are diagnosed at the advanced stage when cancer cells are actively metastasizing, and the 5-year survival rate of patients with advanced OC is only about 30% [4, 5]. Therefore, it is necessary to screen and identify reliable biomarkers to ensure the accuracy of early diagnosis. Besides, understanding the pathogenesis and metastatic mechanisms of OC may help the development of targeted therapies.

Recently, the intriguing role of circRNA (a kind of non-coding RNA) in human diseases has aroused widespread interest in society [6]. Compared with linear precursor mRNA, circRNA is widely distributed and stably expressed because of its covalently closed-loop structure (without 3’ and 5’ ends) [7]. It can be found in exosomes, saliva and plasma [7–9]. Therefore, circRNAs are considered to be more reliable biomarkers or therapeutic targets in cancers. In OC, numerous circRNAs have been functionally explored. For example, circRNA LARP4 was underexpressed in OC tissues and closely linked to the poor prognosis of OC patients [10]. CircRNA CSPP1 overexpression accelerated OC cell proliferation and invasion through mediating miR-1236-3p expression inhibition [11]. A previous study put forward that hsa_circ_0004712 was upregulated in ovarian ectopic endometrium [12]. However, its property in OC remained unclear. It was worth exploring whether hsa_circ_0004712 was also aberrantly expressed in OC and potential functions.

MicroRNA (miRNA) is a class of regulatory molecules with 18 ~ 24 nucleotides [13]. Plenty of miRNAs are mentioned to take part in cancer progression by acting as direct targets of upstream circRNAs or binding to 3’ untranslated region of downstream mRNAs [14]. Following these mechanisms, miR-331-3p was documented to participate in the pathogenesis of various cancers [15, 16]. However, the detailed functions of miR-331-3p and mechanism associated with hsa_circ_0004712 were not fully illuminated.

Frizzled 4 (FZD4) is a member of the frizzled (FZD) family that is well-known as a receptor of the WNT signaling pathway [17]. The dysregulation of FZD4 was mentioned to be associated with the development of human diseases, including cancer [18]. It was reported that FZD4 high expression was associated with high-risk of EOC [19], whereas its functions and regulatory mechanisms in OC were insufficient.

In the current study, the expression level of hsa_circ_0004712 was monitored in OC tissues and cells. Besides, we downregulated hsa_circ_0004712 expression to investigate its role on cell proliferation, apoptosis and metastasis in vitro as well as tumorigenesis in vivo. In addition, we established the link among hsa_circ_0004712, miR-331-3p and FZD4 to present a novel mechanism of hsa_circ_0004712 action in OC and provide evidence for hsa_circ_0004712 as a biomarker.

## Result

### Hsa_circ_0004712 was richly regulated in OC tissues and cells

**Fig. 1:**
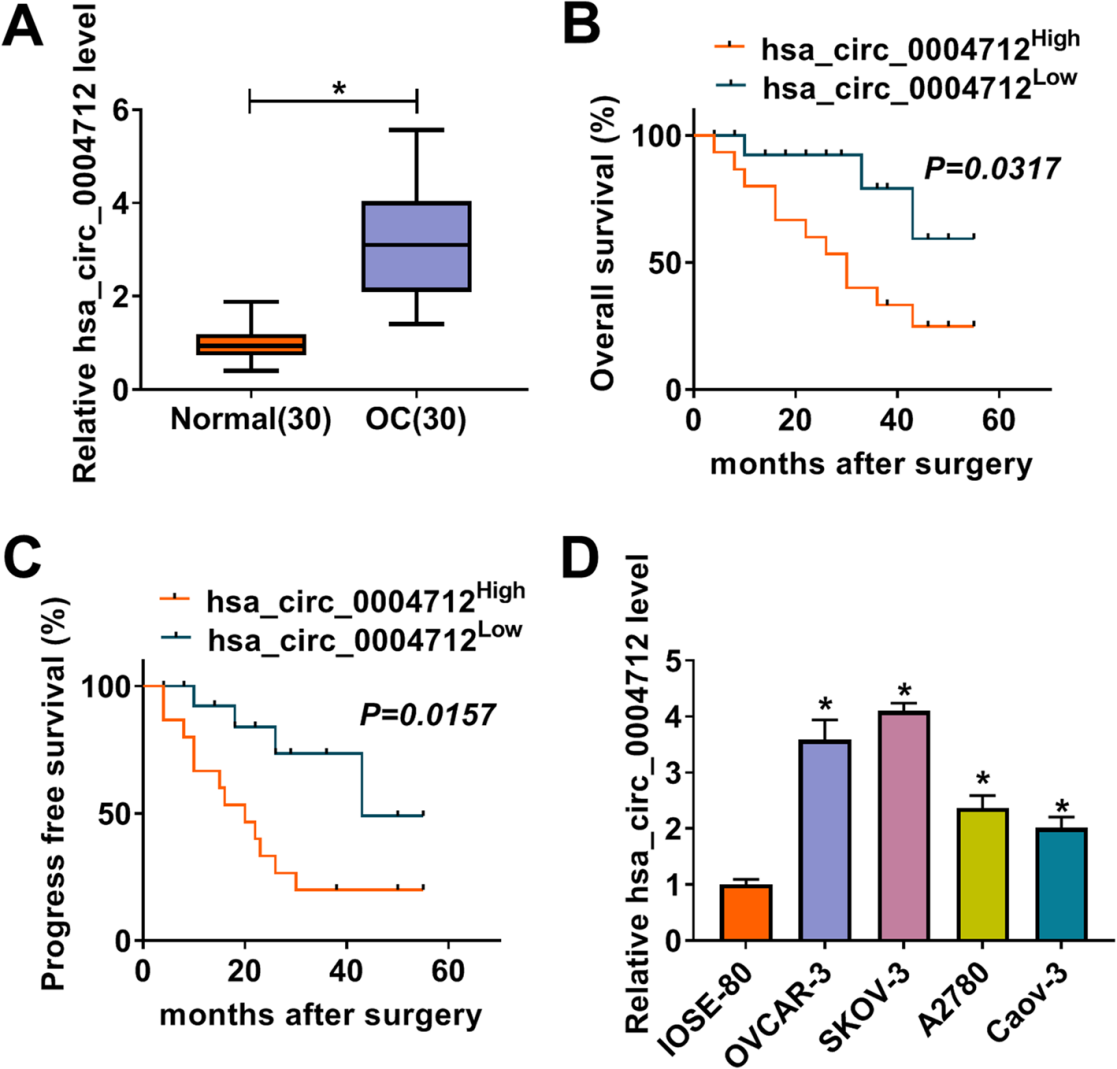
The expression of hsa_circ_0004712 was elevated in OC tissues and cells. **A** Hsa_circ_0004712 expression in OC tissues was detected by qRT-PCR. **B** and **C** The overall survival curve and progression-free survival curve were generated by Kaplan–Meier plot and log-rank test. **D** Hsa_circ_0004712 expression in OC cell lines was detected by qRT-PCR. *P < 0.05

At first, we examined the expression of hsa_circ_0004712 in OC. As shown in Fig. 1A, the abundance of hsa_circ_0004712 in clinical OC tissues (*n* = 30) was notably higher than that in normal tissues (*n* = 30). Besides, the records showed that OC patients with higher hsa_circ_0004712 expression harbored a relatively poor overall survival rate within 5 years (Fig. 1B). In addition, high hsa_circ_0004712 expression was related to low progression-free survival of OC patients within 5 years (Fig. 1C). The data of overall survival and progress free survival associated with miR-331-3p or FZD4 were shown in Fig. S1. The data revealed that high miR-331-3p expression was associated with high overall survival and progress free survival (Fig. S1A and B), and high FZD4 expression was associated with poor overall survival and progress free survival (Fig. S1C and D). Hsa_circ_0004712 was also highly expressed in OC cell lines, including OVCAR-3, SKOV-3, A2780 and Caov-3 cells, compared with that in IOSE-80 cells (Fig. 1D). In a word, the expression of hsa_circ_0004712 was aberrantly upregulated in OC.

### Hsa_circ_0004712 downregulation blocked OC cells proliferation, stimulated apoptosis and attenuated invasion/migration

**Fig. 2:**
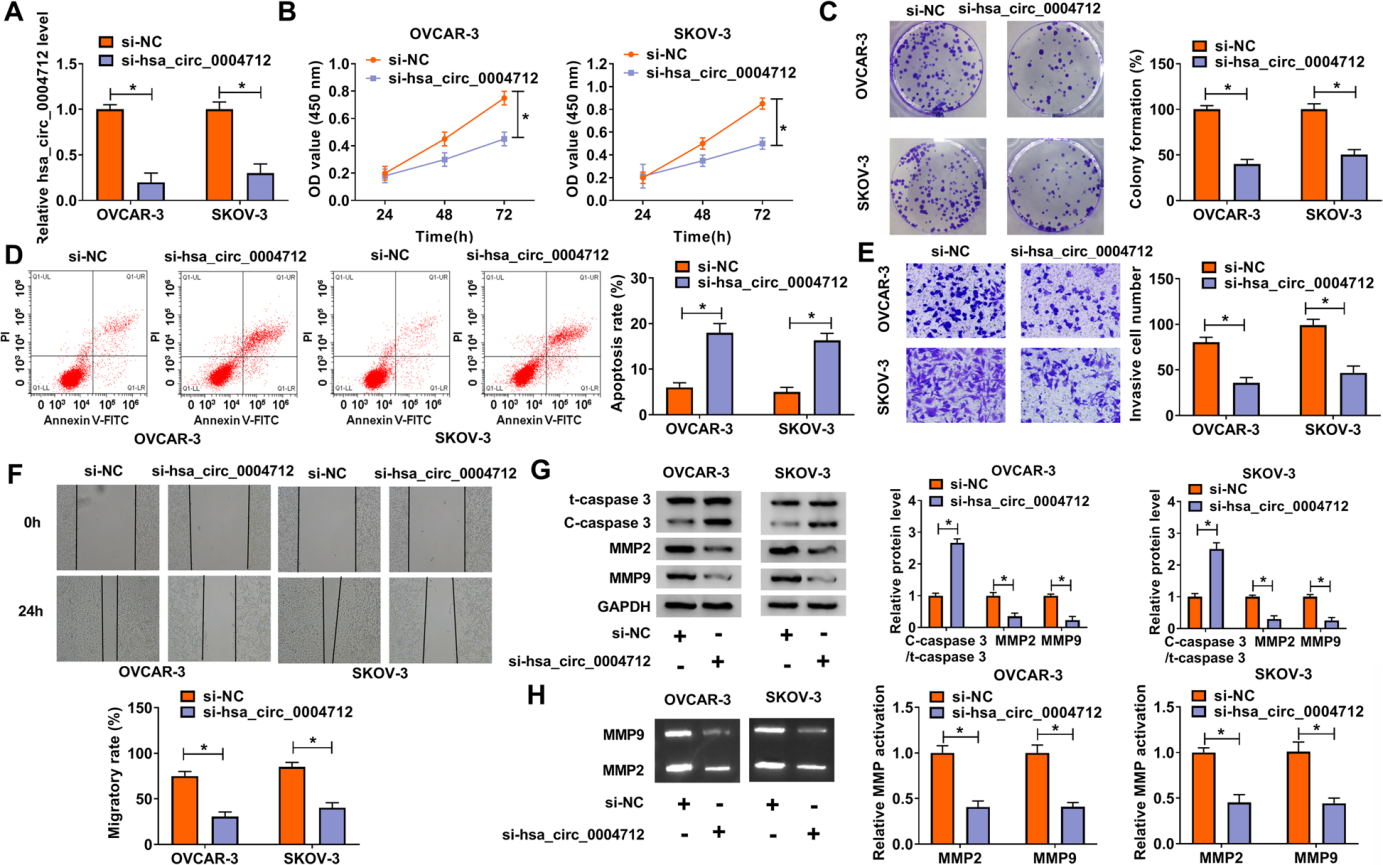
Hsa_circ_0004712 knockdown blocked OC development in vitro. **A** In si- hsa_circ_0004712 transfected cells, the expression of hsa_circ_0004712 was measured by qRT-PCR. **B** and **C** Cell proliferation ability was assessed by CCK-8 assay and colony formation assay. **D** Cell apoptosis was checked by flow cytometry assay. **E** Cell invasion was assessed by transwell assay. **F** Cell migration was assessed by wound healing experiment. **G** The expression of C-caspase, MMP2 and MMP9 was detected by western blot. **H** The activities of MMP2 and MMP9 were determined by gelatin zymography analysis. *P < 0.05

SiRNA targeting hsa_circ_0004712 was inserted into OVCAR-3 and SKOV-3 cells to diminish the expression of hsa_circ_0004712. The qRT-PCR data presented that the expression of hsa_circ_0004712 was significantly declined in cells with si-hsa_circ_0004712 transfection (Fig. 2A). For function analyses, hsa_circ_0004712 downregulation visibly impaired cell proliferation ability as well as colony formation ability by the analyses of CCK-8 assay and colony formation assay (Fig. 2B and C). Inversely, flow cytometry analysis exhibited that hsa_circ_0004712 downregulation signally strengthened the apoptosis rate of OVCAR-3 and SKOV-3 cells (Fig. 2D). Transwell invasion assay showed that hsa_circ_0004712 downregulation significantly reduced the number of invaded cells (Fig. 2E). Wound healing assay hsa_circ_0004712 downregulation remarkably restrained the migratory distance (Fig. 2F). In addition, several invasion/migration-associated markers, including C-caspase 3, MMP2 and MMP9, were quantified, and data hsa_circ_0004712 downregulation significantly strengthened C-caspase 3 level while lessened MMP2 and MMP9 levels (Fig. 2G), suggesting migration and invasion of OVCAR-3 and SKOV-3 cells were suppressed by hsa_circ_0004712 downregulation. Additionally, the activities of MMP2 and MMP9 were strikingly decreased in OVCAR-3 and SKOV-3 cells after hsa_circ_0004712 downregulation (Fig. 2H). These functional analyses indicated that hsa_circ_0004712 downregulation blocked OC cell malignant activities.

### Hsa_circ_0004712 targeted miR-331-3p to suppress its expression

**Fig. 3:**
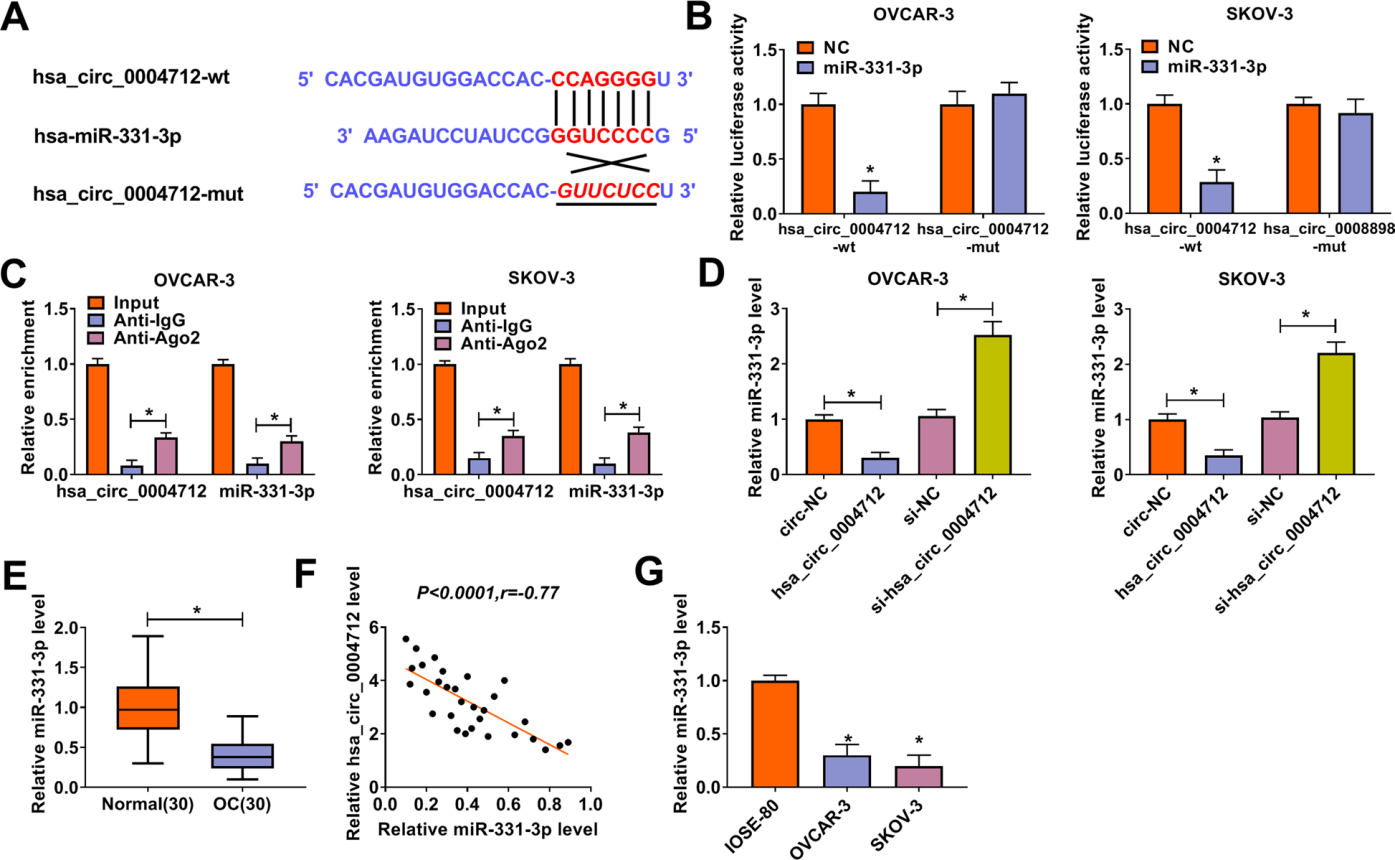
Hsa_circ_0004712 bound to miR-331-3p and repressed miR-331-3p expression. **A** The interaction between hsa_circ_0004712 and miR-331-3p was predicted by CircInteractome. **B** and **C** The interaction between hsa_circ_0004712 and miR-331-3p was verified by dual-luciferase reporter assay and RIP assay. **D** The expression of miR-331-3p was detected in cells with hsa_circ_0004712 overexpression or knockdown. **E** The expression of miR-331-3p in OC tissues. **F** The correlation between hsa_circ_0004712 expression and miR-331-3p expression in OC tissues. **G** The expression of miR-331-3p in OC cell lines. *P < 0.05

MiR-331-3p might be a potential target of hsa_circ_0004712 with a special binding site between it and hsa_circ_0004712 sequences (CircInteractome), and then the mutated sequence of hsa_circ_0004712 (binding site mutation) was designed for dual-luciferase reporter assay (Fig. 3A). Through dual-luciferase reporter analysis, we found that the transfection of miR-331-3p significantly reduced the luciferase activity of hsa_circ_0004712-wt-transfected cells compared with the control, but did not affect the luciferase activity of the cells transfected with hsa_circ_0004712-mut (Fig. 3B). Additionally, RIP assay showed that both hsa_circ_0004712 and miR-331-3p were simultaneously enriched in the anti-Ago2 experimental group compared to that in the anti-IgG control group (Fig. 3C). Moreover, we found that hsa_circ_0004712 overexpression weakened miR-331-3p expression, but hsa_circ_0004712 downregulation increased miR-331-3p expression in OVCAR-3 and SKOV-3 cells (Fig. 3D). All data demonstrated that miR-331-3p was a target of hsa_circ_0004712. We next detected the expression of miR-331-3p in OC tissues and discovered that miR-331-3p was remarkably downregulated in OC tissues (*n* = 30) compared with that in normal tissues (*n* = 30) (Fig. 3E), and further analysis stated that miR-331-3p expression was negatively correlated with hsa_circ_0004712 expression in OC tissues (Fig. 3F). Also, the expression of miR-331-3p was strikingly decreased in OVCAR-3 and SKOV-3 cells compared to IOSE-80 cells (Fig. 3G).

### Hsa_circ_0004712 downregulation regulated cell proliferation, apoptosis, migration and invasion by increasing miR-331-3p expression

**Fig. 4:**
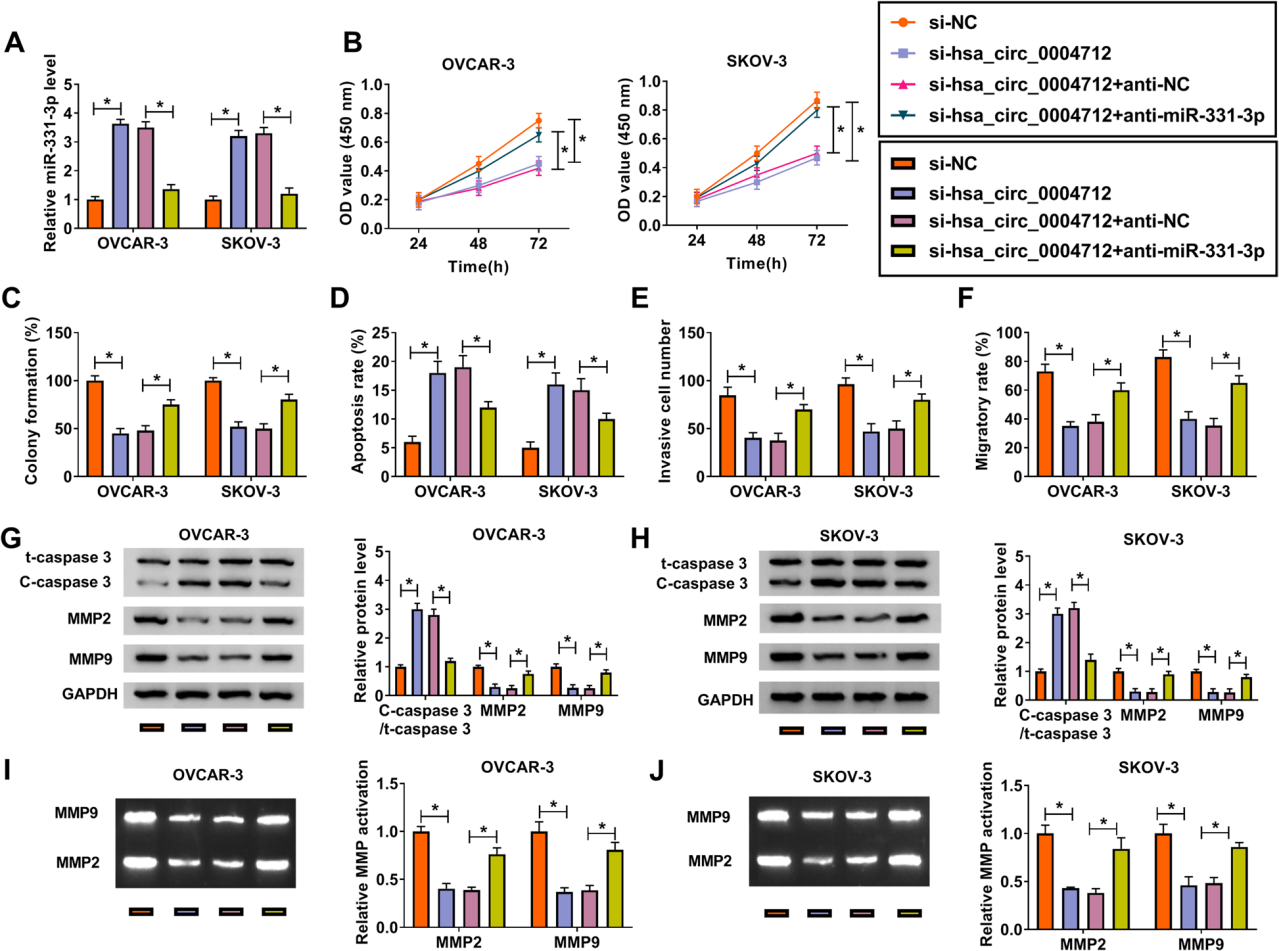
Hsa_circ_0004712 knockdown blocked OC development in vitro by upregulating miR-331-3p. OVCAR-3 and SKOV-3 cells were transfected with si-hsa_circ_0004712, si-NC, si-hsa_circ_0004712 + anti-miR-331-3p or si-hsa_circ_0004712 + anti-NC. **A** The expression of miR-331-3p in these transfected cells. **B** Cell proliferation detected by CCK-8 assay. **C** Colony formation ability detected by colony formation assay. **D** Cell apoptosis detected by flow cytometry. **E** Cell invasion detected by transwell assay. **F** Cell migration detected by wound healing assay. **G** and **H** The expression of C-caspase 3, MMP2 and MMP9 detected by western blot. **I** and **J** The activities of MMP2 and MMP9 were determined by gelatin zymography analysis. *P < 0.05

To figure out whether hsa_circ_0004712 targeted miR-331-3p to mediate its biological functions, we conducted rescue experiments in OVCAR-3 and SKOV-3 cells by transfecting with si-hsa_circ_0004712 and si-hsa_circ_0004712 + anti-miR-331-3p, respectively, si-NC and si-hsa_circ_0004712 + anti-NC serving as separate control. The efficiency of miR-331-3p mimic and inhibitor was checked, and miR-331-3p expression was largely increased in OVCAR-3 and SKOV-3 cells transfected with miR-331-3p but largely decreased in cells transfected with anti-miR-331-3p (Fig. S2A and B). The expression of miR-331-3p was detected to be highly increased in cells transfected with si-hsa_circ_0004712 but decreased in cells transfected with si-hsa_circ_0004712 + anti-miR-331-3p (Fig. 4A). Cell proliferation and colony formation abilities were suppressed by si-hsa_circ_0004712 transfection but promoted by si-hsa_circ_0004712 + anti-miR-331-3p transfection (Fig. 4B and C; Fig. S3A). While the situation of cell apoptosis was opposite to cell proliferation (Fig. 4D; Fig. S3D). As for cell invasion and migration, both of them were impaired in cells transfected with si-hsa_circ_0004712 but reinforced in cells transfected with si-hsa_circ_0004712 + anti-miR-331-3p (Fig. 4E and F; Fig. S3B and C). The expression of C-caspase 3 increased in cells with si-hsa_circ_0004712 transfection was largely decreased in cells with si-hsa_circ_0004712 + anti-miR-331-3p transfection, while the expression of MMP2 and MMP9 was opposite to C-caspase 3 expression (Fig. 4G and H). Besides, the activities of MMP2 and MMP9 depleted by si-hsa_circ_0004712 were partly recovered by the reintroduction of anti-miR-331-3p (Fig. 4I and J). These data suggested that the effects caused by si-hsa_circ_0004712 could be reversed by the reintroduction of anti-miR-331-3p, hinting that hsa_circ_0004712 knockdown played functions by increasing miR-331-3p expression.

### FZD4 was a target of miR-331-3p, and its expression was suppressed by miR-331-3p but strengthened by hsa_circ_0004712

**Fig. 5:**
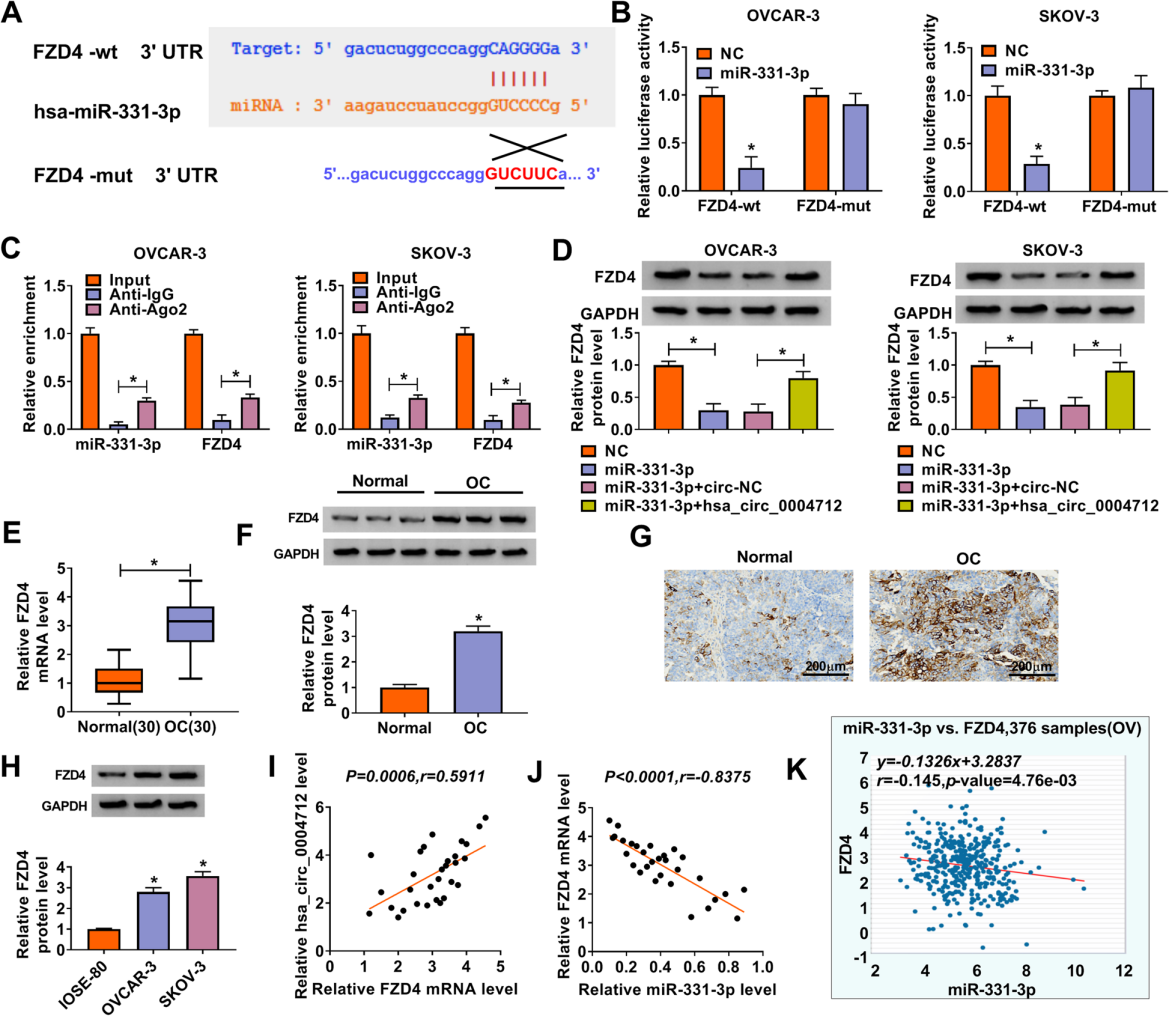
FZD4 was targeted by miR-331-3p and positively regulated by hsa_circ_0004712. **A** The interaction between miR-331-3p and FZD4 was predicted by starBase. **B** and **C** The interaction between miR-331-3p and FZD4 was validated by dual-luciferase reporter assay and RIP assay. **D** The expression of FZD4 in cells transfected with miR-331-3p, NC, miR-331-3p + hsa_circ_0004712 or miR-331-3p + circ-NC was detected by western blot. **E** and **F** FZD4 expression in OC tissues was detected by qRT-PCR and western blot. **G** FZD4 expression in OC tissues was determined by IHC staining analysis. **H** The expression of FZD4 in OC cells was detected by western blot. **I** and **J** The association between FZD4 expression and hsa_circ_0004712 expression or miR-331-3p expression was analyzed by Spearman’s test. **K** The association between FZD4 and miR-331-3p expression was obtained from starBase. *P < 0.05

Further analysis prompted that FZD4 might be a target of miR-331-3p with a special targeting site between its 3’UTR and miR-331-3p sequence by the prediction of starBase (Fig. 5A). As validations, the luciferase activity was markedly reduced in OVCAR-3 and SKOV-3 cells cotransfected with miR-331-3p and FZD4-wt but not FZD4-mut (Fig. 5B), and both of FZD4 and miR-331-3p were abundantly expressed in the Ago2 RIP group compared to the IgG RIP group (Fig. 5C). Moreover, we found that the expression of FZD4 was overtly weakened in cells with miR-331-3p transfection but elevated in cells with miR-331-3p + hsa_circ_0004712 transfection (Fig. 5D), suggesting that hsa_circ_0004712 reintroduction could promote FZD4 expression. In addition, the expression of FZD4 was noteworthily increased in OC tissues and cells compared to normal tissues and non-cancerous cells, respectively (Fig. 5E, F and H). The enhanced expression of FZD4 in OC tissues was also confirmed by IHC staining analysis (Fig. 5G). Furthermore, the expression of FZD4 in OC tissues (*n* = 30) was positively associated with hsa_circ_0004712 expression but negatively associated with miR-331-3p expression (Fig. 5I and J). Interestingly, the data from starBase website also showed a negative correlation between FZD4 and miR-331-3p expression in OC samples (*n* = 376) (Fig. 5K). These data indicated that hsa_circ_0004712 positively regulated FZD4 expression by targeting miR-331-3p.

### MiR-331-3p restoration repressed OC cell proliferation, promoted apoptosis and limited migration and invasion by restricting FZD4

**Fig. 6:**
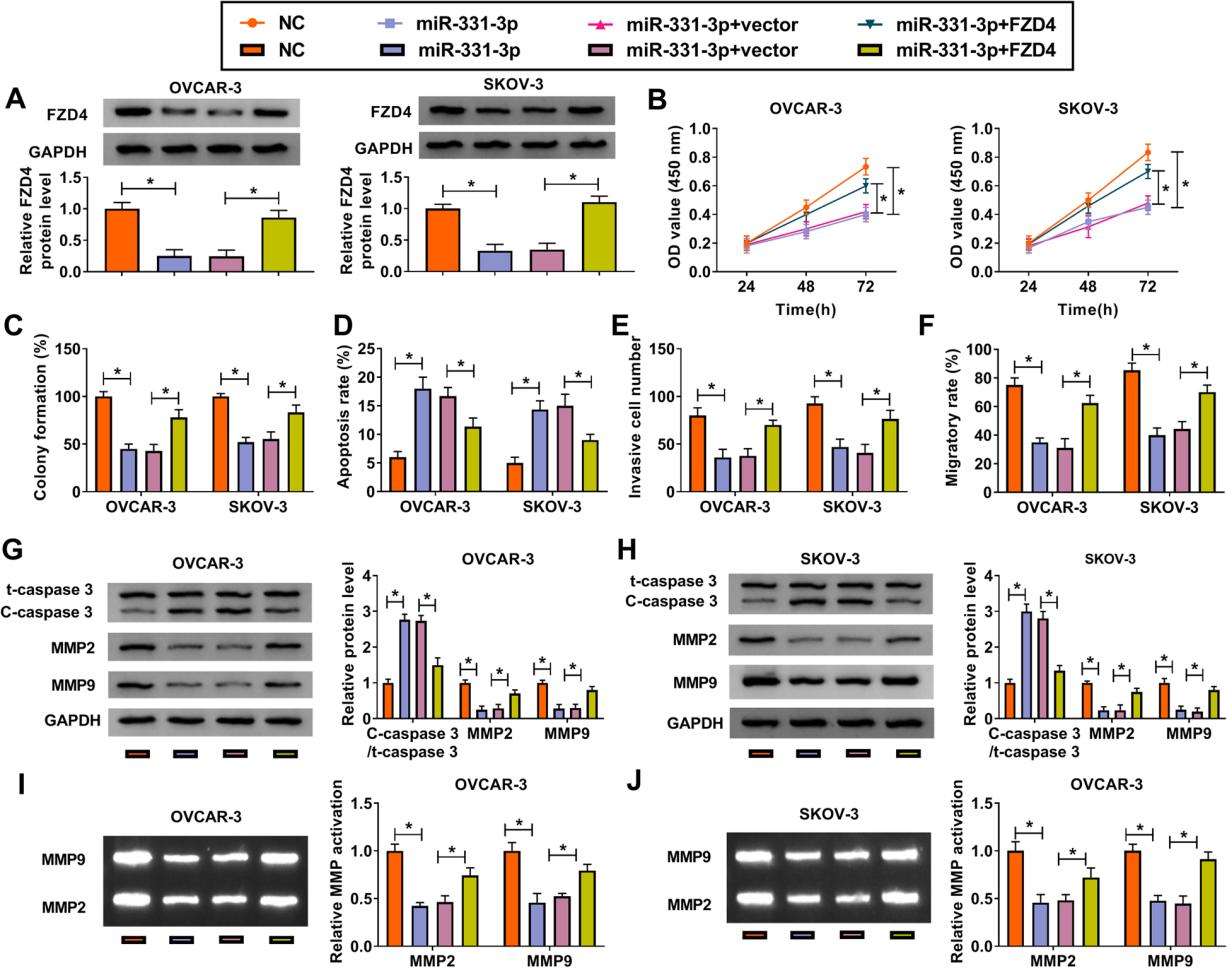
MiR-331-3p played anti-tumor role in OC by suppressing FZD4. OVCAR-3 and SKOV-3 cells were transfected with miR-331-3p, NC, miR-331-3p + FZD4 or miR-331-3p + vector. **A** The expression of FZD4 detected by western blot. **B** Cell proliferation assessed by CCK-8 assay. **C** Colony formation ability assessed by colony formation assay. **D** Cell apoptosis assessed by flow cytometry assay. **E** Cell invasion assessed using transwell assay. **F** Cell migration assessed using wound healing assay. **G** and **H** The expression of C-caspase 3, MMP2 and MMP9 measured by western blot. **I** and **J** The activities of MMP2 and MMP9 were determined by gelatin zymography analysis. *P < 0.05

Next we explored the effects of the interaction between miR-331-3p and FZD4 in OC cells. OVCAR-3 and SKOV-3 cells were introduced with miR-331-3p or miR-331-3p + FZD4, and NC or miR-331-3p + vector was acted as corresponding control. The expression of FZD4 was significantly reduced in cells transfected with miR-331-3p, while FZD4 expression was recovered in cells transfected with miR-331-3p + FZD4 (Fig. 6A). MiR-331-3p restoration notably repressed cell proliferation ability and colony formation ability, while together overexpressed miR-331-3p and FZD4 substantially promoted the abilities of cell proliferation and colony formation (Fig. 6B and C; Fig. S4A). On the contrary, alone miR-331-3p overexpression stimulated cell apoptosis, while concurrent overexpression of miR-331-3p and FZD4 partly blocked cell apoptosis (Fig. 6D; Fig. S4D). The capacities of cell invasion and migration were attenuated in cells transfected with miR-331-3p but intensified in cells transfected with miR-331-3p + FZD4 (Fig. 6E and F; Fig. S4B and C). Additionally, the expression of MMP2 and MMP9 was prominently declined in cells with miR-331-3p transfection but partly restored in cells with miR-331-3p + FZD4 transfection, while the expression of C-caspase 3 was opposite to them (Fig. 6G and H). The activities of MMP2 and MMP9 suppressed by miR-331-3p were partly restored by the reintroduction of FZD4 (Fig. 6I and J). All results indicated that miR-331-3p defended OC cell malignant activities by weakening the expression of FZD4.

### Hsa_circ_0004712 knockdown blocked tumor growth by regulating miR-331-3p and FZD4 expression

**Fig. 7:**
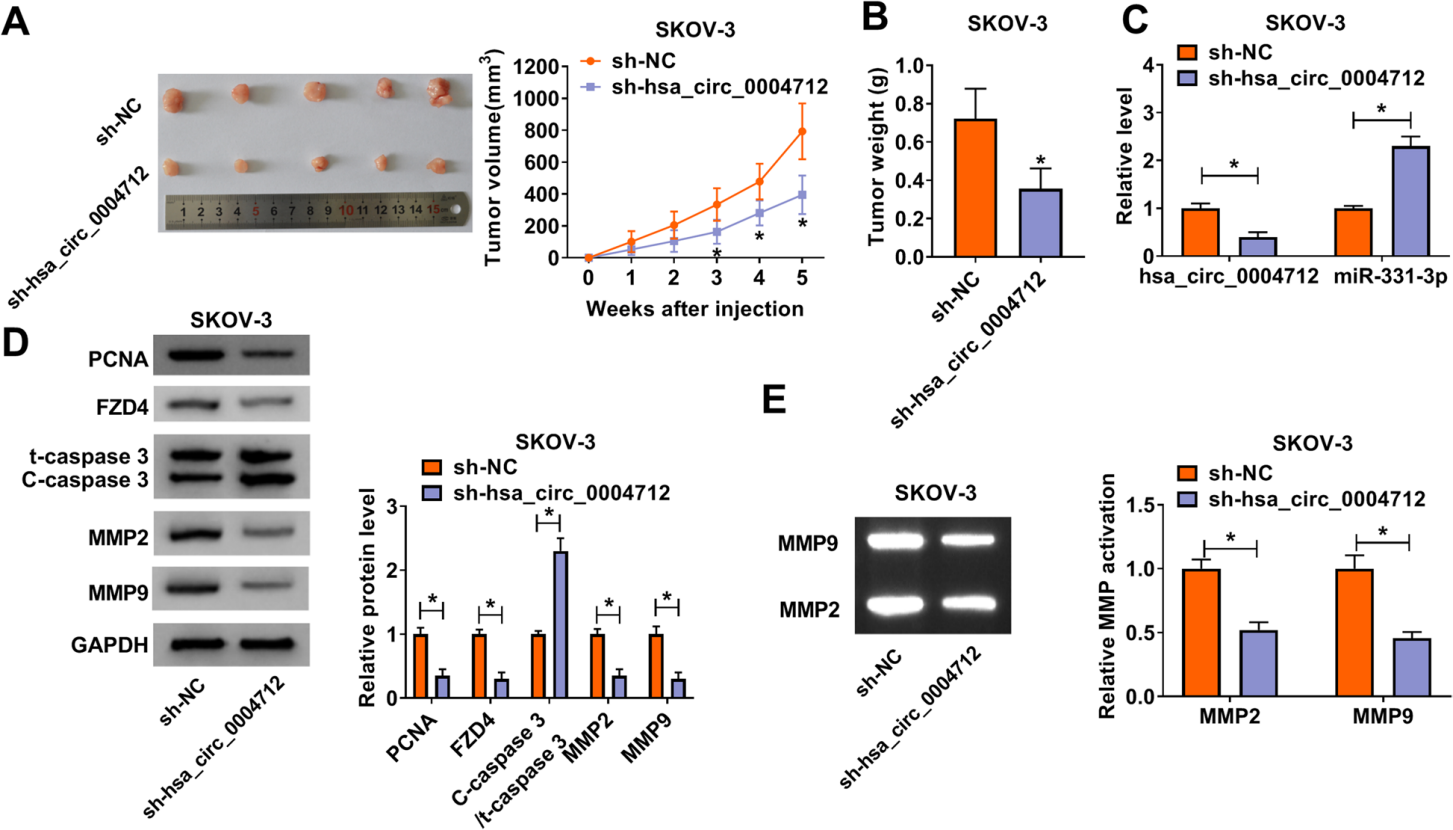
Hsa_circ_0004712 knockdown inhibited tumor growth in vivo. **A** Tumor volume recorded once a week. **B** Tumor weight measured after 5 weeks. **C** The expression of hsa_circ_0004712 and miR-331-3p in removed tissues. **D** The expression of FZD4, PCNA, C-caspase 3, MMP2 and MMP9 in removed tissues. **E** The activities of MMP2 and MMP9 in the removed tissues were determined by gelatin zymography analysis. *P < 0.05

Animal experiments were conducted to test the function of hsa_circ_0004712 in vivo. The experimental data manifested hsa_circ_0004712 knockdown substantially restrained tumor volume and tumor weight (Fig. 7A and B). Besides, we examined the expression of hsa_circ_0004712, miR-331-3p and FZD4 in these samples. The expression of hsa_circ_0004712 was significantly decreased, while miR-331-3p expression was elevated in the sh-hsa_circ_0004712 experimental group compared with that in the sh-NC control group (Fig. 7C). The expression tendency of FZD4 was consistent with hsa_circ_0004712 expression (Fig. 7D). To test the growth characteristics of tumors, several proteins, including PCNA, C-caspase 3, MMP2 and MMP9, were quantified. The data presented that PCNA, MMP2 and MMP9 were all strikingly downregulated in the sh-hsa_circ_0004712 experimental group compared to the sh-NC control group, while the level of C-caspase 3 was opposite to them (Fig. 7D), suggesting that tumor growth was inhibited. In addition, the activities of MMP2 and MMP9 were largely declined in tumor tissues in the sh-hsa_circ_0004712 group (Fig. 7E). Collectively, hsa_circ_0004712 knockdown also limited tumor development in vivo by regulating miR-331-3p and FZD4.

## Discussion

CircRNAs are promising detection tools to monitor cancer development. The establishment of circRNA-related regulatory networks contributes to understanding of cancer pathogenesis. With the boom of RNA sequencing technology, a growing number of circRNAs have been screened and identified [20], which provides numerous differently expressed circRNAs in tumor tissues and non-tumor tissues [21]. Based on it, circRNA functions are widely investigated in cancer cell models, including OC [22]. CircRNA ITCH was strikingly downregulated in OC tissues and functioned as a tumor inhibitor to block OC cell proliferation and migration [23]. While circSMAD7 was observed to be highly expressed in OC tissues and played cancer-promoting roles to force tumorigenesis and metastasis [24]. In our study, we discussed a novel circRNA, hsa_circ_0004712, which was documented to be aberrantly upregulated in ovarian ectopic endometrium by microarray analysis [12]. The expression of hsa_circ_0004712 was detected in clinical samples and cell lines, and higher expression of hsa_circ_0004712 existed in OC tissues and cells. Besides, high expression of hsa_circ_0004712 was closely related to high grade of OC and lymph node metastasis in clinicopathologic features, and high expression of hsa_circ_0004712 was associated with poor overall survival and progression-free survival. The data hinted that hsa_circ_0004712 might be a promising prognostic marker in OC. In function, silencing hsa_circ_0004712 repressed OC cell proliferation, invasion and migration, and hsa_circ_0004712 knockdown also blocked tumor development in vivo, suggesting that hsa_circ_0004712 served as a cancer-promoting role in OC.

Previous studies addressed that circRNAs played anti-tumor or pro-tumor roles by acting as miRNAs “sponges” to activate mRNAs expression [11, 23]. We speculated that hsa_circ_0004712 might also function following this way. The bioinformatics database (CircInteractome) deduced that miR-331-3p was a potential target of hsa_circ_0004712, which was validated by dual-luciferase reporter assay and RIP assay. MiR-331-3p was a well-recognized tumor suppressor in various cancers, including gastric cancer, hepatocellular carcinoma and lung cancer [16, 25, 26]. A recent study also introduced that miR-331-3p was downregulated in OC and restrained OC cell proliferation and metastasis [27]. Consistent with these studies, we found miR-331-3p restoration also repressed cell proliferation, invasion and migration, and miR-331-3p deficiency could reverse the functional effects of hsa_circ_0004712 downregulation. Our study provided a novel mechanism of miR-331-3p action in OC.

Further study determined that FZD4 was bound by miR-331-3p. FZD4 was regarded as one of the signatures to predict the recurrence of OC [28]. Besides, long non-coding RNA HOXD-AS1 promoted the expression of FZD4 by targeting miR-608, thereby accelerating the malignant development of OC [29]. In agreement with these consequences, we concluded that FZD4 was highly expressed in OC tissues and cells, and FZD4 overexpression reversed the anti-tumor effects of miR-331-3p, hinting that FZD4 was an oncogene in OC, which was consistent with the carcinogenic properties of FZD4 in other cancers [30–32].

## Conclusion

Summarily, an increased expression level of hsa_circ_0004712 was observed in OC tissues and cells. Hsa_circ_0004712 positively modulated the expression of FZD4 by targeting miR-331-3p. Hsa_circ_0004712 downregulation suppressed OC malignant development via mediating the miR-331-3p/FZD4 pathway. Our study for the first time monitored the function of hsa_circ_0004712 in OC, which broadened our eyes to realize OC pathogenesis.

## Methods

### OC tissues

A total of 30 pairs of OC tissues and paired adjacent noncancerous epithelial tissues with no malignant properties were obtained from OC patients during the radical surgery at Shenyang Women’s and Children’s Hospital. The clinicopathological characteristics of these OC patients were shown in Table 1. These specimens were “snap-frozen” in liquid nitrogen and preserved at –80℃. All patients provided written informed consent, and this study protocol was authorized by the Ethics Committee of the Shenyang Women’s and Children’s Hospital.

**Table 1:** Correlation of the expression of hsa_circ_0004712, miR-331-3p, FZD4 and clinicopathologic features in patients with ovarian cancer

Parameters		hsa_circ_0004712 expression		*P*-value		miR-331-3p expression		*P*-value		FZD4 expression		*P*-value
	*N* = 30	High *N* = 15	Low *N* = 15		*N* = 30	High *N* = 15	Low *N* = 15		*N* = 30	High *N* = 15	Low *N* = 15	
Age, years												
< 60	16	7	9	0.464	16	6	10	0.143	16	8	7	0.858
≥ 60	14	8	6		14	9	5		14	7	7	
Tumor size												
< 4 cm	19	9	10	0.705	19	8	11	0.256	19	7	12	0.058
≥ 4 cm	11	6	5		11	7	4		11	8	3	
Histological classification												
Serous	20	9	11	0.439	20	12	8	0.121	20	11	9	0.439
Mucinous	10	6	4		10	3	7		10	4	6	
Grade												
G1	12	3	9	0.025*	12	8	4	0.136	12	2	10	0.003*
G2 + G3	18	12	6		18	7	11		18	13	5	
FIGO stage												
< III	13	4	9	0.065	13	8	5	0.26	13	3	10	0.010 *
≥ III	17	11	6		17	7	10		17	12	5	
Lymph node metastasis												
Yes	19	13	6	0.008*	19	7	12	0.058	19	12	7	0.017*
No	11	2	9		11	8	3		11	2	9	
Chemosensitivity												
Yes	12	5	7	0.456	12	7	5	0.456	12	4	8	0.136
No	18	10	8		18	8	10		18	11	7	

^*^P < 0.05, representing statistical significance

### Cell lines

Human OC cell lines (OVCAR-3 and SKOV-3) and normal ovarian epithelial cells (IOSE-80) were bought from BeNa Culture Collection (Beijing, China). OVCAR-3 cells were maintained in 90% Dulbecco’s Modified Eagle Medium (DMEM; Gibco, Grand Island, NY, USA) plus 10% fetal bovine serum (FBS; Gibco), and SKOV-3 and IOSE-80 cells were maintained in 90% Roswell Park Memorial Institute 1640 (RPMI 1640; Gibco) medium containing 10% FBS. All cells were cultured in a 37℃ incubator containing 5% CO_2_.

### Oligonucleotides, plasmids, and transfection

Small interference RNA specially targeting hsa_circ_0004712 (si-hsa_circ_0004712) for hsa_circ_0004712 downregulation, short hairpin RNA (shRNA) lentiviral vector containing hsa_circ_0004712, and their corresponding controls (si-NC or sh-NC) were loaded by GeneCopoeia (Guangzhou, China). MiR-331-3p mimics (miR-331-3p), miR-331-3p inhibitors (anti-miR-331-3p) and their controls (NC and anti-NC) were obtained from Ribobio (Guangzhou, China). CircRNA overexpression vector (pCD25-ciR) containing hsa_circ_0004712 for hsa_circ_0004712 overexpression (hsa_circ_0004712), pcDNA vector containing FZD4 for FZD4 overexpression (FZD4), and their separate control (circ-NC and vector) were constructed by GeneSeed (Guangzhou, China). All of them were transfected into OVCAR-3 and SKOV-3 cells using Lipofectamine 3000 Reagent (Invitrogen, Carlsbad, CA, USA).

### Quantitative real-time polymerase chain reaction (qRT-PCR)

After obtaining total RNA using TRIzol reagent (Invitrogen), reverse transcription reactions were conducted using the PrimeScript RT reagent kit (TaKaRa, Dalian, China) following the manufacturer’s protocol for hsa_circ_0004712 and FZD4. Real-time PCR was then carried out using the SYBR Premix Ex Taq II (Takara) under a 7500 System (Applied Biosystems, Foster City, CA, USA). Glyceraldehyde-3-phosphate dehydrogenase (GAPDH) was used to normalize their expression. The expression of miR-331-3p was detected using the miRNA qRT-PCR analysis kit (Ribobio) in line with the directions. U6 RNA was used to normalize its expression. The primer sequences were: hsa_circ_0004712, F: 5’-AGGGGTGAACCAGCCATTT-3’ and R: 5’-GCCAATCTCCCCTGAGTATGTT-3’, FZD4, F: 5’-AGCTCGTGCCCAACCAGGTT-3’ and R: 5’-ATGCCGCCGCATGGGCCAAT-3’, GAPDH, F: 5’-CAATGACCCCTTCATTGACC-3’ and R: 5’-GACAAGCTTCCCGTTCTCAG-3’. miR-331-3p, F: 5’-GAGCTGAAAGCACTC CCAA-3’ and R: 5’-CACACTCTTGATGTTCCAGGA-3’, U6, F: 5’-ACACTCCAGCTGGGTGAGATGAAGCACTGTAG-3’ and R: 5’- CTCAACTGGTGTCGTGGA-3’. All results were calculated using the 2^−ΔΔCt^ method and presented as the fold change relative to respective controls.

### Cell counting kit-8 (CCK-8)

OVCAR-3 and SKOV-3 cells with transfection were loaded into 96-well plates (5 × 10^3^ cells/ well). After 24, 48 or 72 h, 10 μL CCK-8 solution (KeyGen Biotech, Nanjing, China) was added into each well for another 2 h at 37 ℃ followed by the detection of absorbance at 450 nm using a microplate reader (Bio-Rad, Hercules, CA, USA).

### Colony formation assay

OVCAR-3 and SKOV-3 cells with transfection were seeded into 6-well plates in triplicate to allow colony growth. After 12 days of incubation, plates were softly rinsed with phosphate-buffered saline (PBS) and stained with 0.1% crystal violet. Colony forms were next observed and counted under a microscope (Nikon, Tokyo, Japan).

### Flow cytometry assay

OVCAR-3 and SKOV-3 cells with transfection were plated into 6-well plates and cultured for 48 h. Subsequently, cells were washed with PBS and exposed to trypsin. Cells were measured by double staining with Annexin V/Propidium Iodide (PI) from the Apoptosis Assay Annexin V and PI kit (Biotium, Hayward, CA, USA) for 30 min in the dark. The stained cells were analyzed by flow cytometry (BD Bioscience, San Jose, CA, USA).

### Transwell invasion assay

OC cells with transfection were incubated for 24 h. Meanwhile, transwell chambers (Corning Incorporated, Corning, NY, USA) were coated with Matrigel (Corning Incorporated) at 4℃ overnight. Then, cells were transferred into the upper chamber of transwell chambers coated with Matrigel. After 24 h, non-invaded cells were gently discarded with a cotton swab, and invaded cells in the lower surface were immobilized and stained with 0.1% crystal violet. The invasion phenotype was observed under a microscope (Nikon).

### Wound healing assay

OC cells with different transfection were seeded into 6-well plates and incubated for 24 h. An artificial wound was created onto the monolayer using a 10 μL pipette tip. Allowing wound healing for 24 h, cells were rinsed with serum-free culture medium, and images of cell migration were photographed under an inverted microscope (100 × ; Nikon). Mitomycin C (Sigma-Aldrich, St. Louis, MO, USA) was added for pretreatment to eliminate the effect of cell proliferation on cell migration.

### Western blot

Tissues or cells were lysed using RIPA buffer (KeyGen Biotech) to obtain total proteins. After quantification, equal proteins (20 μg) were loaded on 10% sodium dodecyl sulfate–polyacrylamide gel electrophoresis (SDS-PAGE) for separation. Polyvinylidene difluoride (PVDF; Bio-Rad) membranes with separated proteins were treated with the blocking buffer containing 5% skim-milk. After that, membranes were incubated with the primary antibodies at 4℃ overnight, including anti-total caspase 3 (anti-t-caspase 3; ab13847; Abcam, Cambridge, MA. USA), anti-cleaved caspase-3 (anti-C-caspase 3; ab2302; Abcam), anti-metalloproteinase 2 (anti-MMP 2; ab92536; Abcam), anti-metalloproteinase 9 (anti-MMP 9; ab76003; Abcam), anti-FZD4 (ab83042; Abcam), anti-proliferating cell nuclear antigen (anti-PCNA; ab92552; Abcam) and anti-GAPDH (ab9485; Abcam), and the secondary antibody (goat anti-rabbit; ab502718; Abcam) at 25℃ for 1 h. The bands were viewed using the ECL (enhanced chemiluminescence) Detection Kit (KeyGen Biotech).

### Gelatin zymography analysis

The activities of MMP 2 and MMP 9 in the culture medium were determined by gelatin zymography assay. Cells with different transfections were cultured in complete medium at 37℃ for 6 h. Then, cells were collected and placed in a serum-free medium to continue culturing for 24 h. Without boiling or reducing, the culture supernatant was then separated by electrophoresis using an 8% polyacrylamide gel containing 1.5 mg/mL gelatin (Sigma-Aldrich). After electrophoresis, the gels were washed with 2.5% Triton X-100 and incubated for 12 h in reaction buffer (40 mM Tris–HCl, pH 8.0, 10 mM CaCl2) at 37℃. Subsequently, the gels were stained with 0.5 mg/mL Coomassie Brilliant Blue R-250 (Sigma-Aldrich) and washed with 5% methanol and 10% acetic acid mixed decolorizing solution.

### Bioinformatics analysis

Databases CircInteractome (https://circinteractome.nia.nih.gov/) and starBase (http://starbase.sysu.edu.cn/) were employed to analyze the targets and binding sites.

### Dual-luciferase reporter assay

The luciferase reporter plasmids were generated using the pGL4 vector containing a wild-type or mutant fragment of hsa_circ_0004712, which harbored wild-type or mutant miR-331-3p binding site. The fusion plasmids were named as hsa_circ_0004712-wt or hsa_circ_0004712-mut. Similarly, FZD4-wt (pGL4 containing wild-type fragment of FZD4) and FZD4-mut (pGL4 containing a mutant fragment of FZD4) were also constructed. OVCAR-3 and SKOV-3 cells were transfected with the abovementioned reporter plasmids and miR-331-3p or NC, respectively. After 48 h, assays for luciferase activity detection were performed using the Dual-Luciferase Reporter Assay System (Promega) after 48 h of transfection.

### RNA immunoprecipitation (RIP) assay

The EZ-Magna RIP Kit (Millipore, Billerica, MA, USA) was utilized to further test the interaction between miR-331-3p and hsa_circ_0004712 or FZD4. First, cells were lysed in RIP lysis buffer, and cell extract was then incubated with RIP buffer mixing with magnetic beads conjugated with anti-Argonaute 2 (anti-Ago2) antibody (Millipore) or anti-Immunoglobulin G (IgG) antibody (control; Millipore). After incubation with proteinase K, the immunoprecipitated RNA was isolated to subject to qRT-PCR analysis.

### Immunohistochemical (IHC) analysis

5-μm-thickness tissue sections were prepared, followed by dewaxing and blocking. Subsequently, tissues sections were incubated with the primary antibody targeting FZD4 (ab83042) at 4℃ overnight and next incubated with the secondary antibody for 1 h at room temperature. Finally, tissue sections were stained using a 3, 3’-diaminobenzidine (DAB) kit (Abcam) and observed under a microscope.

### Animal experiments

All procedures were conducted with the approval of the Institutional Animal Care and Use Committee of Shenyang Women’s and Children’s Hospital. BALB/c mice (4–6 weeks old, female) were purchased from Shanghai Model Organisms (Shanghai, China) with regular housing conditions. SKOV-3 cells (5 × 10^6^) that stably expressed sh-hsa_circ_0004712 or sh-NC were subcutaneously injected into the right flank of the mice. Each group contained six mice. Tumor volume (length × width^2^ × 0.5) was measured once a week, lasting five weeks. At the end of tumor growth, all mice were sacrificed, and the tumor tissues were removed for the following analyses.

### Statistical analysis

All assays contained at least three separate experiments. Statistical analyses were handled using GraphPad Prism 7.0 software (La Jolla, CA, USA). Spearman’s correlation test was used to analyze the association between miR-331-3p and hsa_circ_0004712 or FZD4 expression in tumor tissues. Kaplan–Meier survival plots were generated with log-rank statistics to monitor the survival rate. Difference comparison was conducted using Student’s *t*-test between two groups or using analysis of variance plus Tukey test among multiple groups. All data were shown as mean ± standard deviation. *P* < 0.05 was considered statistically significant.

## Supplementary Information

**Additional file 1: Fig. S1.** The association between miR-331-3p expression or FZD4 expression and overall survival and progress free survival.

**Additional file 2: Fig. S2.** The efficiency of miR-331-3p mimic and inhibitor in OVCAR-3 and SKOV-3 cells.

**Additional file 3: Fig. S3.** The representative images of colony formation, wound healing, transwell and flow cytometry assays in OVCAR-3 and SKOV-3 cells transfected with si-hsa_circ_0004712 or si-hsa_circ_0004712+anti-miR-331-3p.

**Additional file 4: Fig. S4.** The representative images of colony formation, wound healing, transwell and flow cytometry assays in OVCAR-3 and SKOV-3 cells transfected with miR-331-3p or miR-331-3p+FZD4.

## Data Availability

All data generated or analyzed during this study are available from the corresponding author on reasonable request.

## Abbreviations

circRNAs: Circular RNAs
OC: Ovarian cancer
EOC: Epithelial ovarian cancer
miRNA: MicroRNA
FZD4: Frizzled 4
FZD: Frizzled
