# Supplementary figures and images for: Hsa_circ_0004712 downregulation attenuates ovarian cancer malignant development by targeting the miR-331-3p/FZD4 pathway
Source: J Ovarian Res. 2021 Sep 10;14:118. doi: 10.1186/s13048-021-00859-0 (PMC8434704; doi:10.1186/s13048-021-00859-0)

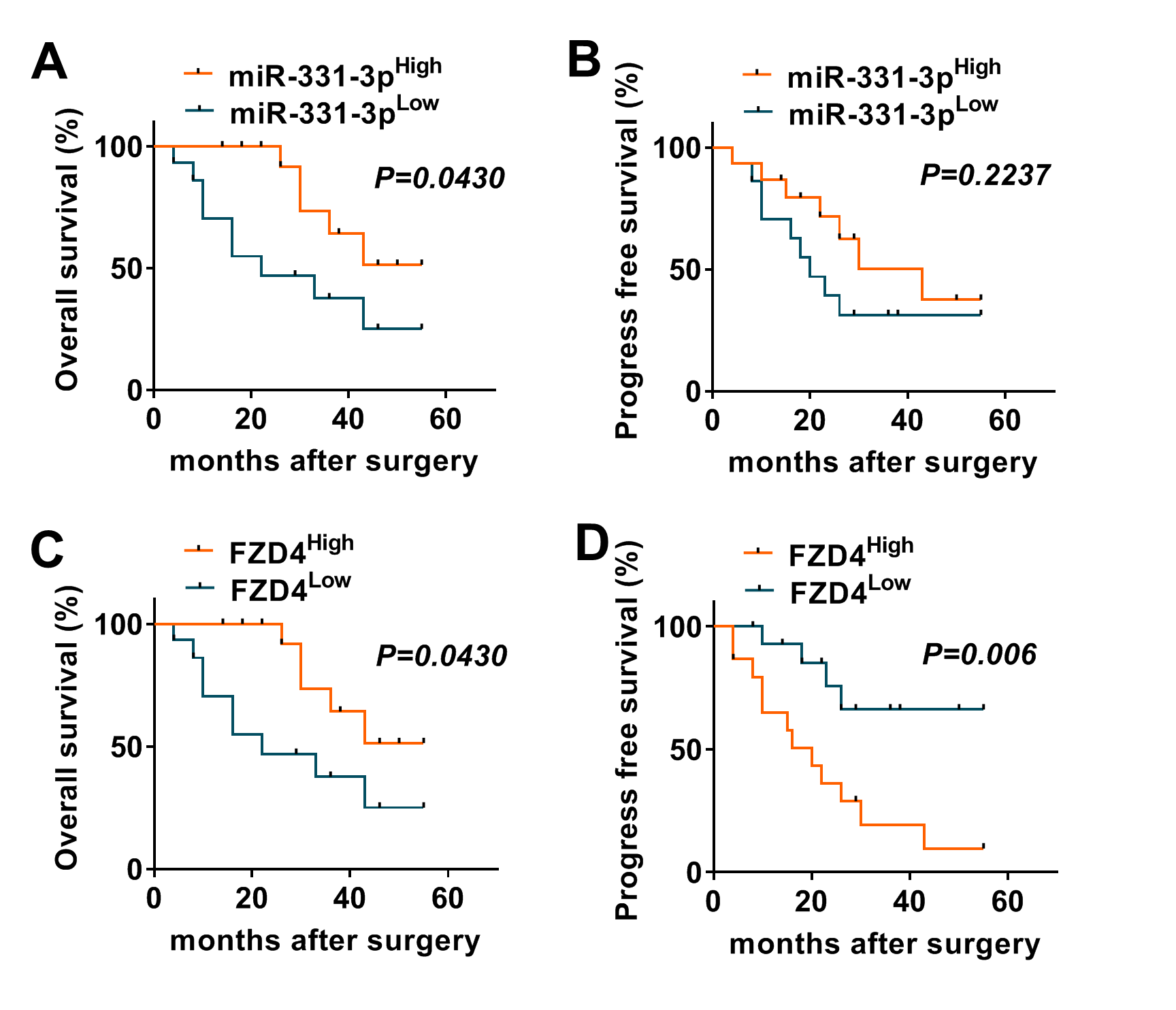

Supplement: Supplementary file 1 — Additional file 1: Fig. S1. The association between miR-331-3p expression or FZD4 expression and overall survival and progress free survival. [file 13048_2021_859_MOESM1_ESM.tif]

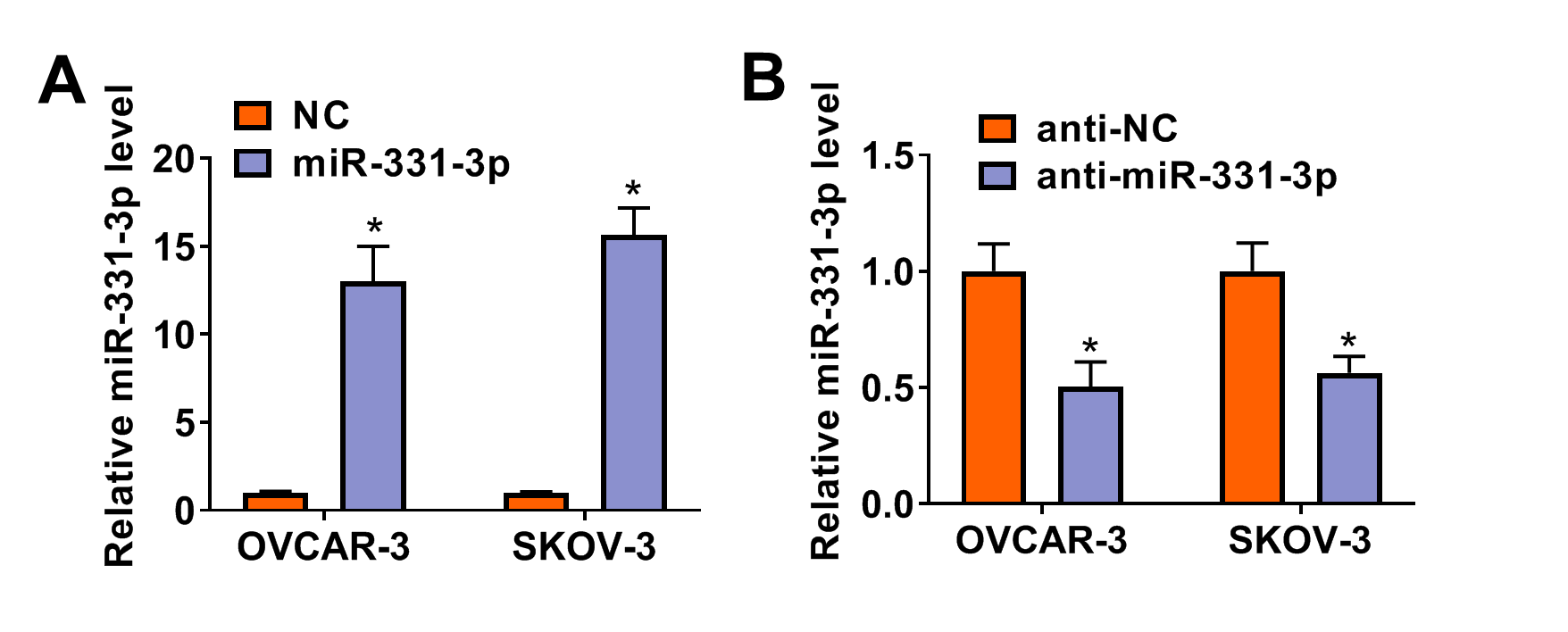

Supplement: Supplementary file 2 — Additional file 2: Fig. S2. The efficiency of miR-331-3p mimic and inhibitor in OVCAR-3 and SKOV-3 cells. [file 13048_2021_859_MOESM2_ESM.tif]

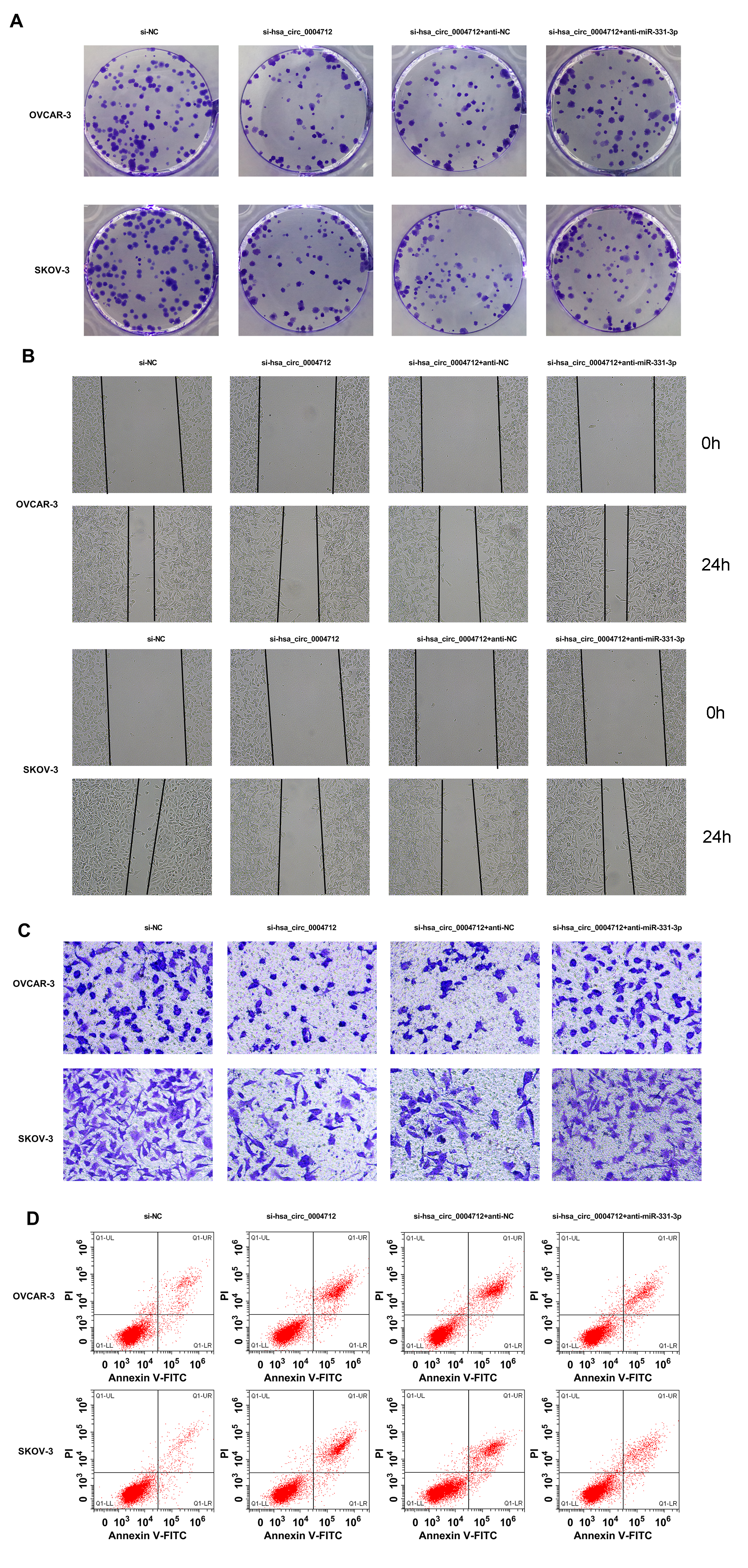

Supplement: Supplementary file 3 — Additional file 3: Fig. S3. The representative images of colony formation, wound healing, transwell and flow cytometry assays in OVCAR-3 and SKOV-3 cells transfected with si-hsa_circ_0004712 or si-hsa_circ_0004712+anti-miR-331-3p. [file 13048_2021_859_MOESM3_ESM.tif]

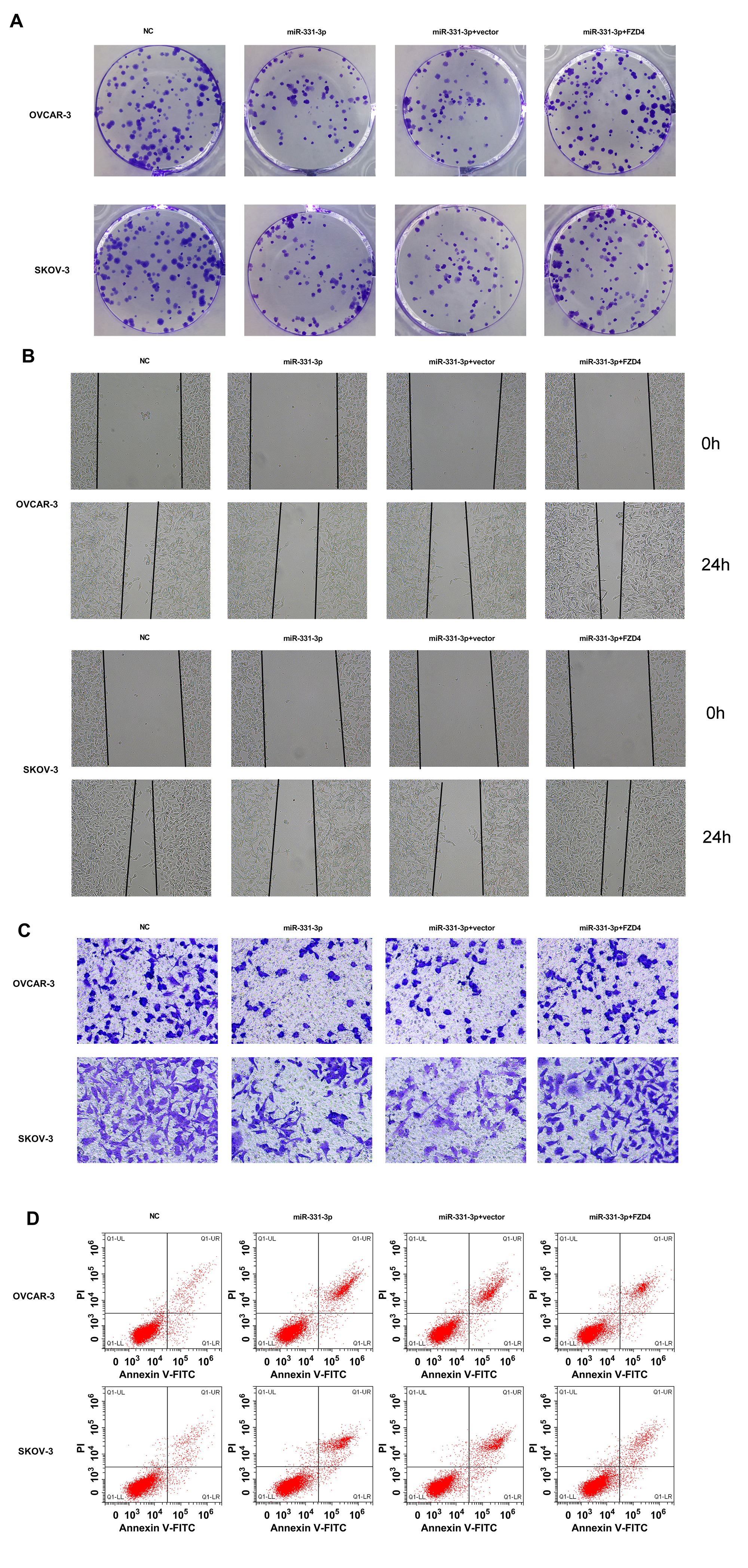

Supplement: Supplementary file 4 — Additional file 4: Fig. S4. The representative images of colony formation, wound healing, transwell and flow cytometry assays in OVCAR-3 and SKOV-3 cells transfected with miR-331-3p or miR-331-3p+FZD4. [file 13048_2021_859_MOESM4_ESM.tif]
